# Vitamin E and lycopene reduce coal burning fluorosis-induced spermatogenic cell apoptosis via oxidative stress-mediated JNK and ERK signaling pathways

**DOI:** 10.1042/BSR20171003

**Published:** 2018-07-31

**Authors:** Yuan Tian, Yuehai Xiao, Bolin Wang, Chao Sun, Kaifa Tang, Fa Sun

**Affiliations:** Department of Urology, Affiliated Hospital of Guizhou Medical University, Guiyang 550004, P. R. China

**Keywords:** apoptosis, fluorosis, lycopene, oxidative stress, testis, vitamin E

## Abstract

Although fluoride has been widely used in toothpaste, mouthwash, and drinking water to prevent dental caries, the excessive intake of fluoride can cause fluorosis which is associated with dental, skeletal, and soft tissue fluorosis. Recent evidences have drawn the attention to its adverse effects on male reproductive system that include spermatogenesis defect, sperm count loss, and sperm maturation impairment. Fluoride induces oxidative stress through the activation of mitogen activated protein kinase (MAPK) cascade which can lead to cell apoptosis. Vitamin E (VE) and lycopene are two common antioxidants, being protective to reactive oxygen species (ROS)-induced toxic effects. However, whether and how these two antioxidants prevent fluoride-induced spermatogenic cell apoptosis are largely unknown. In the present study, a male rat model for coal burning fluorosis was established and the histological lesions and spermatogenic cell apoptosis in rat testes were observed. The decreased expression of clusterin, a heterodimeric glycoprotein reported to regulate spermatogenic cell apoptosis, was detected in fluoride-treated rat testes. Interestingly, the co-administration with VE or lycopene reduced fluorosis-mediated testicular toxicity and rescued clusterin expression. Further, fluoride caused the enhanced Jun N-terminal kinase (JNK, c-Jun) and extracellular signal-regulated protein kinase (ERK) phosphorylation, which was reduced by VE or lycopene. Thus, VE and lycopene prevent coal burning fluorosis-induced spermatogenic cell apoptosis through the suppression of oxidative stress-mediated JNK and ERK signaling pathway, which could be an alternative therapeutic strategy for the treatment of fluorosis.

## Introduction

Fluoride exits naturally in soil, water, and food at varying amount and is required for normal development and growth of animals. Fluoride is critical in maintaining the metabolism of calcium and phosphorus in body; however, excessive intake of fluoride can cause fluorosis, a serious public health problem associated with dental mottling and skeletal manifestations. Additionally, excessive fluoride intake can also induce damage to soft tissues including liver, kidney, brain, and testis [1]. Particularly, recent evidences have drawn much attention to the adverse effects of fluoride on male reproductive system. Those include a defect in spermatogenesis [2,3], a reduction in sperm count [2,4,5], and the impairment on the differentiation and maturation of spermatozoa [6]. Cell apoptosis is one early sign of genotoxic damage in mature testis, and plays critical roles in spermatozoa. Fluoride harbors various cellular effects and excessive fluoride may cause oxidative stress, inhibit protein secretion and transport [7,8], induce inflammatory response [9,10], and interfere with cell proliferation and migration which could be fluoride concentration and/or cell type dependent [8,11,12]. However, the underlying mechanisms of these cellular functions are less clear.

Oxidative stress is generally recognized as a model of fluoride action and observed in a set of cells including pancreatic β-cells and neuronal cells [13,14] and in soft tissues such as liver, brain, lung, and testes. Fluoride inhibits the activity of antioxidant enzymes, such as superoxide dismutase (SOD), changes the level of antioxidant glutathione (GSH), and causes excessive production of reactive oxygen species (ROS) in mitochondrion, leading to lipid peroxidation and cell apoptosis. It has been reported that fluoride induces oxidative stress through the activation of mitogen activated protein kinase (MAPK) signaling pathways by the phosphorylation of Jun N-terminal kinase (JNK) and extracellular signal-regulated protein kinase (ERK), leading to the modulation of cell growth, differentiation, and apoptosis [15,16].

Vitamin E (VE) and lycopene are two common antioxidants in daily drink and diet. The protective effects of VE and lycopene on fluoride-induced toxic effects have been revealed. The co-administration of VE has been shown to protect testicular disorders in male rats [17,18]. Lycopene supplement can attenuate fluoride-induced oxidative stress and caspase pathway thereby reducing fluoride-induced ameloblasts apoptosis and dental fluorosis [19]. Whether and how VE and lycopene regulate fluorosis-induced oxidative stress and spermatogenic cell apoptosis in testes are less clear. The present study established the rat model for coal burning fluorosis and investigated the effect of fluorosis on spermatogenic cell apoptosis and oxidative stress in rat testes. The co-administration of VE or lycopene suppressed fluorosis-induced spermatogenic cell apoptosis in rat testes via the modulation of oxidative stress-mediated JNK and ERK signaling pathway.

## Materials and methods

### Animals and administration of fluoride

Male Sprague–Dawley rats were provided by the Experimental Animal Center of Guiyang Medical College, Guiyang, China. Sixty rats (6–8 weeks old; weighing 90–110 g) were randomly divided into six groups (five rats/group) as follows: a vehicle-treated group; low-fluoride group feeding with 20 mg/kg fluoride-containing standard chows; moderate-fluoride group feeding with 40 mg/kg fluoride-containing standard chows; high-fluoride group feeding with 60 mg/kg fluoride-containing standard chows; high fluoride and 40 mg/kg VE co-treated group; and high fluoride and 40 mg/kg lycopene co-treated group. To mimic the fluoride poisoning of patients in coal-burning regions, rats in fluoride-treated groups were fed with standard chows mixed with corn dried by burning coal from endemic fluorosis areas to reach required amount of fluoride (20, 40, or 60 mg/kg). Treatment was continued for 120 days. The rats were then killed and the testes were dissected. The testis tissue samples were fixed in formalin and embedded in paraffin. Urine samples were collected from each group and the fluoride concentration was measured using ion selective electrode method [20]. In brief, urine samples were collected and incubated at 40°C for 30 min. 1 ml sample was mixed with 0.1 ml ionic strength adjustment buffer. The electrode potential was determined by a pH/ISE meter (Thermos Fisher, US) for calculating urinary fluoride concentration based on calibration slope. The present study was approved by the Animal Care Welfare Committee of Guizhou Medical University.

### Reagents and antibodies

All chemicals or reagents used in the present study were purchased from Sigma (St Louis, MO, USA) unless otherwise indicated. Anti-culsterin was purchased from Boster Biological Technology (Wuhan, China), and anti-GAPDH antibody was purchased from Cell Signaling (Boston, MA, US). All other antibodies including anti-JNK, anti-p-JNK, anti-c-Jun, anti-p-c-Jun, anti-ERK, anti-p-ERK, and anti-β-actin were from Cell Signaling Technology (Danvers, MA, USA).

### Hematoxylin and eosin (H&E) staining

The paraffin-embedded testis were cut into small pieces (5 μm thickness). Following deparaffinization and rehydration, the sections were stained with hematoxylin and eosin and then imaged using a microscopy (Nikon ECLIPSE TS100). Three sections per sample were determined.

### Immunohistochemistry for the detection of clusterin

The prepared testis sections (5 μm thickness) were alternatively blocked with 5% (v/v) bovine normal serum in phosphate-buffered saline. The sections were then incubated with specific antibody against clusterin isoform 75–80 kDa (1:400) overnight at 4°C followed by the incubation with biotinylated secondary antibody for 1 h at room temperature and peroxidase conjugated streptavidin. The sections were treated with 3,3′-diaminobenzidine (Roche, Germany) and subsequently counterstained using hematoxylin. Samples were visualized under a microscope (Nikon ECLIPSE TS100). Three sections per sample were determined.

### TUNEL staining

Terminal deoxynucleotidyl transferase-mediated dUTP nick end labeling (TUNEL) assays were performed using a TUNEL kit (Roche, Mannheim, Germany) following the manufacturer’s instructions. The apoptotic cells were quantified in six random fields in each sample and the corresponding apoptosis index (AI) was calculated.

### Detection of NO, MDA, and GSH

The testes were homogenized in saline on ice followed by centrifugation at 590 x *g* for 10 min at 4°C. The supernatant was then collected for the measurement of NO, MDA, and GSH contents using a commercial NO, MDA, and GSH assay kit (Nanjing Jiancheng Bioengineering Institute, Nanjing), respectively.

### Measurement of NOS, iNOS, and T-SOD activities

The testes homogenate was alternatively used for the determination of nitric oxide synthase (NOS), inducible NOS (iNOS), and T-SOD activities using a NOS, iNOS, and T-SOD kit (Nanjing Jiancheng Bioengineering Institute, Nanjing), respectively.

### Western-blot analysis

Rat testes were homogenized in radio immunoprecipitation assay (RIPA) buffer (25 mmol/l Tris–HCl (pH 7.6), 150 mmol/l NaCl, 1% NP-40, 1% sodium deoxycholate) containing 0.01% protease and phosphatase inhibitors (Roche, Germany). The samples were then centrifuged at 12 000 x *g* for 15 min at 4°C followed by the collection of supernatant. Protein levels were quantified by a BCA Protein Assay Kit (Thermo Fisher Scientific, USA). Protein lysates were separated by 12% sodium dodecyl sulfate-polyacrylamide gel electrophoresis (SDS-PAGE) and transferred onto nitrocellulose membranes (Millipore). Membranes were incubated with relevant antibodies at 4°C overnight followed by horseradish peroxidase (HRP)-conjugated secondary antibody. Membranes were then exposed to ECL reagent (Thermo Fisher Scientific, USA) and the chemiluminescence was detected by Olympus BH-2 Microscopic image acquisition system (Olympus, Japan). β-actin was used as a loading control.

### Statistical analysis

Data shown here are either representative or mean +/± s.e.m. for five rats per group. Data were analyzed by GraphPad Prism 6.0 (GraphPad Software Inc., San Diego, CA) and statistical analysis was performed using one-way or two-way analysis of variance (ANOVA) withTurkey’s multiple comparisons. *P*<0.05 was considered significant. *P<0.05; **P<0.01; ***P<0.001.

## Results

### VE and locopene reduce urinary fluoride in the established rat model for coal burning fluorosis

**Figure 1:**
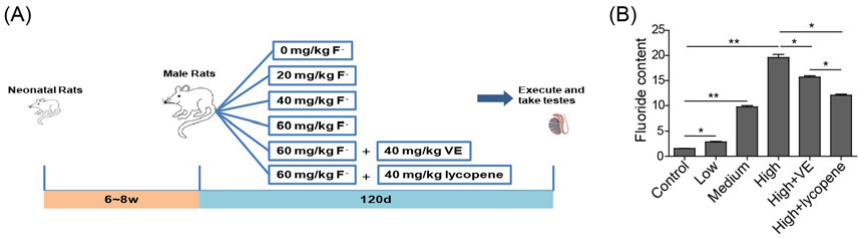
The established rat model for coal burning fluorosis (**A**) The scheme of fluoride treatment in rat. (**B**) The level of fluoride in rat urine under indicated conditions. *n* = 5 of each group. *P<0.05; **P<0.01.

To investigate the role of VE and lycopene on the modulation of fluorosis-induced toxic effects, a coal burning fluorosis model of rats was established. Each group of rats were administrated with vehicle (control), fluoride at low (20 mg/kg), medium (40 mg/kg), or high (60 mg/kg) amount, or co-administrated with high level of fluoride (60 mg/kg) and VE (40 mg/kg) or lycopene (40 mg/kg) (Figure 1A). The observation of fluoride in rat urine indicated the successful construction of this model. The level of fluoride in the urine increased with the increase of fluoride administrated and the high level of fluoride administration resulted into the maximum amount of fluoride detected in urine (Figure 1B, *P*<0.01 comparing to control group). This was successfully inhibited by the co-administrated with VE or lycopene, with more profound effect observed for lycopene. This suggests that VE and lycopene can reduce fluorosis.

### VE and lycopene reduce fluorosis-induced testis damage and cell apoptosis in coal burning fluorosis rats

**Figure 2:**
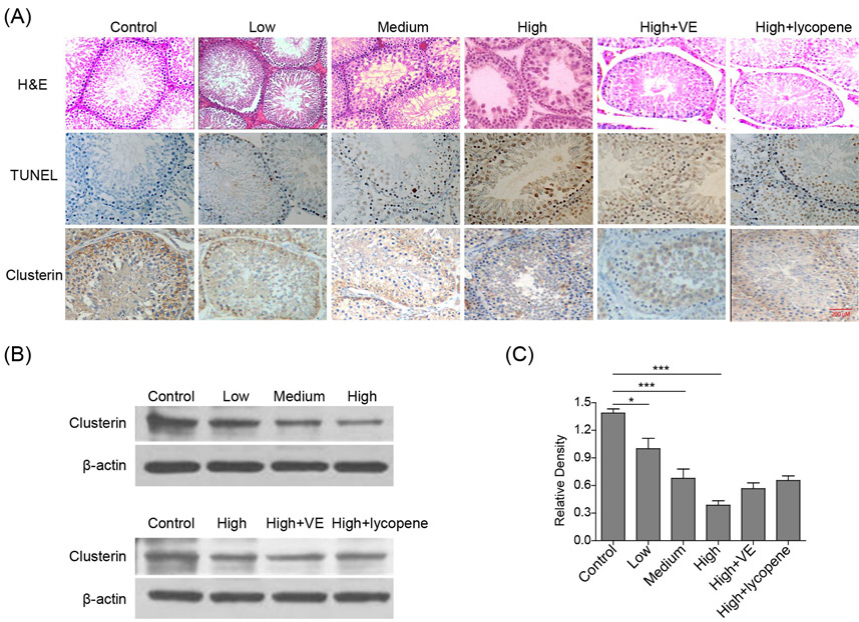
VE and lycopene reduce histological lesions and germ cell apoptosis and rescue clusterin reduction induced by fluoride in rat testes (**A**) Representative image showing H&E, TUNEL, or clusterin staining of rat testes. (**B**) Representative image of Western-blot showing the expression of clusterin in rat testes under indicated conditions. β-actin was used as a loading control. (**C**) The statistical analysis of data in B was presented. *n* = 6 of each group. *P<0.05; ***P<0.001.

Fluoride-induced histological lesions in rat testes were then investigated by H&E staining. In fluoride-treated rats, impaired seminiferous tubular morphology, reduced and disorganized spermatogenic cells, cell swelling, and some cell denature and death were observed (Figure 2A). Particularly, the level of damage enhanced with the increase of administrated fluoride dose. Interestingly, the co-treatment with VE or lycopene prevented fluoride-mediated damage to seminiferous tubular. To further determine fluoride-induced cell apoptosis in rat testes, TUNEL assay was then performed. The AI of seminiferous tubule cells in each fluoride-treated group dose-dependently increased compared with the control (Figure 2A). Consistently, the co-administration with VE or lycopene significantly reduced the AI, indicating the protective effect of VE and lycopene in fluorosis.

The present study further demonstrated the effect of clusterin on spermatogenic cell apoptosis and investigated the mechanism of VE and lycopene to regulate apoptosis. Consistent with the previous reports, the expression of clusterin was reduced in fluoride-treated rat testes, which was partially rescued by the co-treatment with VE or lycopene (Figure 2A–C). This is accordant with the results obtained from TUNEL assay. Collectively, fluorosis induces histological lesion and spermatogenic cell apoptosis in testes, which can be protected by the co-treatment with VE or lycopene.

### VE and lycopene reduce coal burning fluorosis-induced oxidative stress in rat testis

**Figure 3:**
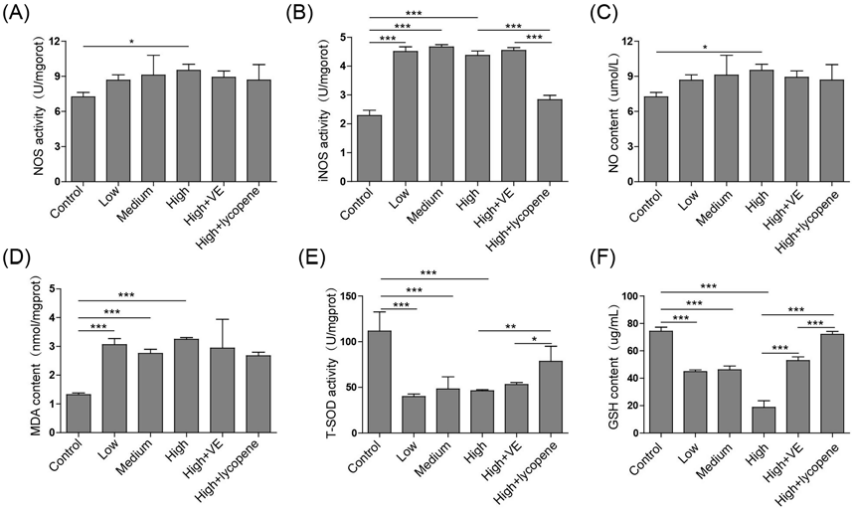
VE and lycopene reduce fluorosis-induced oxidative stress The activities of NOS (**A**), iNOS (**B**), and T-SOD (**E**) and the contents of NO (**C**), MDA (**D**), and GSH (**F**) in rat testis were measured. *n* = 5 of each group. *P<0.05; **P<0.01; ***P<0.001.

Since nitric oxide (NO) availability as a marker for oxidative stress, the level of NO and the activities of NOS and iNOS were measured. As shown in Figure 3A–C, the expression of NO and the activities of NOS and iNOS were elevated in all fluoride-treated groups compared with those in vehicle-treated one, indicating that fluorosis induced oxidative stress in rat testes. Although VE and lycopene had little effect on the expression of NO (Figure 3C), they successfully reduced the increased NOS activity in 60 mg/kg fluoride-treated rat testes (Figure 3A). Interestingly, lycopene markedly suppressed the fluoride-induced increase of iNOS activity whereas VE failed to do so (Figure 3B). Malondialdehyde (MDA) is a marker for oxidative stress and lipid peroxidation. Treatment with fluoride induced the increased expression of MDA suggesting that fluorosis may cause lipid peroxidation in rat testes (Figure 3D, *P*<0.001 comparing to control group). The level of MDA was reduced by co-treatment with VE or lycopene. All these demonstrate the protective effects of VE and lycopene on fluorosis-induced oxidative stress and lipid peroxidation.

To evaluate the antioxidant effect of VE and lycopene, the total SOD (T-SOD) activity and GSH level were determined (Figure 3E,F). The treatment with fluoride induced the decreased T-SOD activity and the reduced GSH content in rat testes. The reduced T-SOD activity induced by 60 mg/kg fluoride was not obviously influenced by the co-administration with VE but was rescued by lycopene (Figure 3E, *P*<0.01 comparing to fluoride group). Both VE and lycopene significantly prevented the fluoride-induced decrease of GSH level and particularly lycopene rescued GSH content to the comparable level as control (Figure 3F, *P*<0.001 comparing to fluoride group).These data imply that VE and lycopene have antioxidant effects and prevent fluorosis-induced testis damage and cell apoptosis by the reduction of fluorosis-mediated oxidative stress.

### VE and lycopene inhibit coal burning fluorosis-induced JNK and ERK phosphorylation

**Figure 4:**
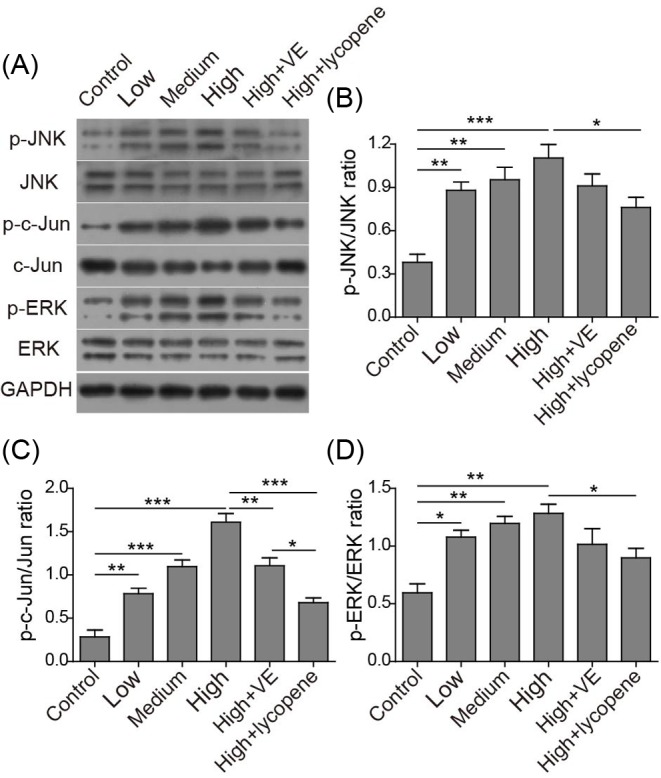
VE and lycopene reduce JNK, c-Jun, and ERK phosphorylation Representative image of Western-blot (**A**) and the corresponding statistical analysis of p-JNK vs. total JNK (**B**), p-c-Jun vs. total c-Jun (**C**), and p-ERK vs. total ERK (**D**). *P<0.05; **P<0.01; ***P<0.001.

Here, we examined the activation of MAPK cascade family members in testis tissues and found that fluoride-treated rats showed elevated expressions of phosphor-JNK (p-JNK), phosphor-c-Jun (p-Jun), and phosphor-ERK (p-ERK) compared with the vehicle-treated one, which was attenuated by the co-treatment with VE or lycopene (Figure 4). This indicates that VE and lycopene reduce fluorosis-induced cell apoptosis by suppressing oxidative stress-mediated JNK and ERK signaling pathway.

## Discussion

Compelling evidences have showed that fluorosis is also associated with the impairment of spermatogenesis and sperm motility. Several lines of studies demonstrate the apoptotic effect of fluoride in brain and metabolic organs whereas the impact of fluoride on spermatogenic cell apoptosis in testes are less clear [1]. The present study further indicates that fluoride can induce damage to seminiferous tubular morphology and spermatogenic cell apoptosis, which are related to fluoride-mediated oxidative stress. These findings are consistent with the previous report [21], which further presented caspase 3 activation, chromatin condensation, DNA fragmentation, death receptor Fas up-regulation, and cytochrome *c* elevation as the mechanisms of fluoride-induced germ cell apoptosis [21]. Together with our observation on the involvement of MAPK in fluoride-mediated cell apoptosis, fluoride is proved to induce cell apoptosis through both intrinsic and extrinsic pathways. Moreover, the correlation between clusterin and spermatogenic cell apoptosis present in the current study further indicate the key role of clusterin in male reproductive system.

Clusterin is also considered as a sensor of oxidative stress [22] and implicated in many cellular functions including lipid transportation, cell survival, and apoptosis [23]. Three major isoforms of clusterin has been described. Among those one nuclear-located 49 kDa form may facilitate apoptosis, and another 75–80 kDa isoform secreted into extracellular matrix mainly suppress cell apoptosis. This study investigated the latter 75–80 kDa isoform. The increased secretion of clusterin is reported to prevent heat stress-induced testicular apoptosis [24]. Studies have demonstrated that fluorosis induces oxidative stress and therefore results into the impairment of cellular redox homeostasis and lipid peroxidation [14,25,26]. The present study consistently demonstrates the decreased antioxidant effect induced by fluorosis, which is possibly related to the reduced clusterin expression. Furthermore, NaF can activate the oxidative stress-mediated MAPK signaling pathway by the phosphorylation of JNK and ERK [15]. It is well known that JNK and ERK are two family members of MAPK essential for the regulation of cell growth and apoptosis. JNKs are transcription factors regulating the expression of pro-apoptotic genes and activating apoptotic signaling pathway. Evidences have shown that ROS can cause apoptosis via JNK phosphorylation [16,27]. ERK can promote apoptosis through multiple mechanisms which include the activation of caspase-8 and the stimulation of cytochrome *c* release [28]. Our finding further confirmed coal burning fluorosis-mediated oxidative stress through JNK and ERK signaling pathways, which might be essential in fluorosis-induced impairment of reproductive system.

Mounting evidences have demonstrated the antioxidant activities of VE and lycopene and presented their protective effects under fluoride exposure. The present study further emphasized that VE and lycopene are antioxidative and can reduce fluorosis-induced oxidative stress and cell apoptosis through the suppression of JNK and ERK phosphorylation. This provides an alternative therapeutic strategy for the treatment of fluorosis-induced toxicity and especially male reproductive system damage. Thus, it is worth to further investigate other molecular mechanisms that regulate fluorosis-mediated oxidative stress and cell apoptosis.

## Abbreviations

AI: apoptosis index
ERK: extracellular signal-regulated protein kinase
iNOS: inducible NOS
JNK: Jun N-terminal kinase
MAPK: mitogen activated protein kinase
MDA: malondialdehyde
NO: nitric oxide
NOS: NO synthase
ROS: reactive oxygen species
SOD: superoxide dismutase
VE: Vitamin E
